# Effectiveness of virtual reality in cancer patients undergoing chemotherapy. Systematic review

**DOI:** 10.1002/ijc.35258

**Published:** 2024-11-16

**Authors:** Jorge Juan Alvarado‐Omenat, Rocío Llamas‐Ramos, Daniel García‐García, Marta Correyero‐León, Emilio Fonseca‐Sánchez, Inés Llamas‐Ramos

**Affiliations:** ^1^ FisioSport Salamanca, S.L. Salamanca Spain; ^2^ Department of Nursing and Physiotherapy Universidad de Salamanca Salamanca Spain; ^3^ Instituto de Investigación Biomédica de Salamanca (IBSAL) Salamanca Spain; ^4^ Faculty of Health Sciences University of Valladolid Soria Spain; ^5^ CRA La Villa Valladolid Spain; ^6^ University Hospital of Salamanca Salamanca Spain

**Keywords:** anxiety, chemotherapy, oncology, pain, virtual reality

## Abstract

Virtual reality is on the rise and is currently postulated as one of the most innovative and promising techniques in the management of pain and anxiety in cancer patients, in the face of painful processes or the stress involved in chemotherapy treatment. The objective has been to find out the effectiveness of virtual reality in patients undergoing chemotherapy. Several literature reviews were conducted between November 2023 and January 2024 in the Pubmed, Web of Science and PEDro databases. The keywords “virtual reality,” “cancer,” “oncology,” “exercise” and “chemotherapy” were combined using the Boolean operator AND. 641 manuscripts were selected as potential manuscripts and after elimination of duplicates and application of the inclusion and exclusion criteria, six articles comprised the final review sample. Virtual reality has proven to be an effective technique in reducing the anxiety, pain, asthenia and stress suffered by patients diagnosed with cancer and chemotherapy treatment. The distraction generated by this therapeutic modality, with a wide range of scenarios, helps to reduce the painful perception and worry of these procedures. However, there are no standard application guidelines or application protocols that demonstrate the superiority of one technique over another. Virtual reality could be a valid complementary tool in the treatment of patients undergoing chemotherapy, showing positive results in pain reduction, anxiety, stress or asthenia. More studies are needed, with larger sample sizes and long‐term follow‐ups to establish treatment protocols in relation to the frequency, intensity, duration and periodicity of interventions with virtual reality.

## Graphical abstract

What's new?

Virtual reality as a therapeutic modality holds significant promise for helping cancer patients manage pain and anxiety. Guidelines on application, duration, and other factors, however, are lacking, and little is known about which specific protocols are the most effective. Here, the authors performed literature reviews to investigate the effectiveness of various virtual reality interventions carried out in patients during chemotherapy sessions. Analyses indicate that overall, virtual reality helps relieve anxiety, pain, asthenia, and stress and is a viable complement to conventional therapies. Additional research is needed to identify virtual reality treatment protocols that provide the greatest benefit for cancer patients.

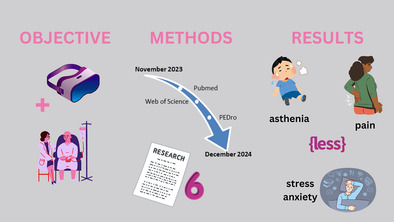

## 1 INTRODUCTION

Virtual reality has been booming in recent years and is currently one of the most innovative and promising techniques.^1^ This therapeutic modality has managed to improve the effectiveness of various clinical interventions, from psychological to pain relief treatments, with satisfactory results for patients.^2^ Specifically, virtual reality is defined as “a simulated experience, consisting of the creation, through hardware and software technology, of virtual environments capable of generating sensations of real experiences”.^3^ Virtual reality manages to provoke a state of distraction, stimulating the brain and influencing the cognitive processes of attention, focusing it on more pleasurable processes, even using scenarios that facilitate the change from a stressful situation such as chemotherapy treatment to another scenario that provides more pleasurable or positive sensations for the user or patient who uses them.^4^

Cancer continues to emerge as one of the leading causes of death and represents a major barrier to increasing life expectancy in all countries of the world^5^ due to aging, population growth, as well as to economic development.^5^ According to the World Health Organization (WHO), cancer is between the first and second leading cause of death before the age of 70 in 112 out of 183 countries, while in 23 other countries it is the third or fourth leading cause of death before the age of 70. Cancer's mortality has increased its importance and has decreased its death prevalence from other diseases such as stroke and heart disease.^6^ The International Agency for Research on Cancer estimated that in 2020, some 19.3 million new cases had been diagnosed worldwide and almost 10 million deaths,^7^ and it is estimated that, by 2040, the number of new cases of cancer pathology will be close to 30 million.^8^ While mortality worldwide is increasing, in Europe it has decreased in recent years (although not for all types of tumors), specifically in Spain, cancer in 2021 represented one of the main causes of morbidity and mortality with an estimated number of new cancers around 276.239.^9^ All this increase in incidence means a great cost for the Spanish health system.^10^

Cancer patients experience many symptoms derived from the disease itself, as well as from their treatments, considerably affecting their quality of life.^11^ There are several scales or instruments for the measurement and knowledge of these symptoms to establish adequate and individualized treatments. The literature reflects scales that assess a single symptom such as the Brief Pain Inventory,^12^ the Karnofsky Performance Status,^13^ the Brief Fatigue Inventory^14^ or scales and questionnaires that assess several symptoms. This group includes the European Organization for Research and Treatment of Cancer Quality of Life Questionnaire Core 30 (EORTC QLQ‐C30),^15^ the Rotterdam Symptom Checklist (RSCL),^16^ or the Memorial Symptom Assessment Scale (MSAS).^17^ Among all of them, the MSAS, in its validated Spanish version,^18^ establishes the prevalence, severity and stress of 32 different symptoms that cancer patients may have experienced in the week prior to assessment, and showed that patients experience an average of 11.09 symptoms during chemotherapy treatments.^19^

As mentioned above, chemotherapy is one of the most common cancer treatments. Cancer and their treatments are accompanied by a series of symptoms such as reduced quality of life, loss of social relationships, fear of death, and anxiety. Anxiety is present in approximately 10% of patients during chemotherapy sessions.^20^ Fatigue in chemotherapy patients reaches 60%–90%^21^ while pain is present in 90%.^22^ There are numerous publications that address the management of pain and anxiety in cancer patients, in the face of painful processes or the stress involved in chemotherapy treatment using virtual reality techniques.^23^ These studies conclude that this technique generates an alteration in the perception of time, making chemotherapy sessions more bearable and tolerable, with positive satisfaction on the part of the patients who have experienced it from the moment the session ends, which lasts up to 48 hours later.^23^, ^24^ Although there are inconclusive results, virtual reality techniques could have an influence on the reduction of vomiting caused by chemotherapy treatment.^23^, ^25^

Virtual reality applications have been extended from symptom management to patient information and management of the concerns this pathology has. In this sense, Greene et al.^26^ created an application with an educational program on “breast cancer, pain and prevention”; this application not only focuses on medical information but also elements of personal history to bring the patient closer to its contents. Most publications deal with outpatients and the reduction and/or elimination of symptoms, but do not focus on patients requiring hospitalization due to this pathology.^27^ Oyama et al.^25^ developed the “Bedside Wellness System” to generate relaxation and positive emotions by hiking in a virtual forest in the beginning and later expanded with a lake and a village, in which sounds, the effect of wind and even aromas could be perceived. Unfortunately, it has only been tested for the relief of symptoms such as fatigue, emotional distress and even vomiting caused by chemotherapy with no definitive results so far.

Despite all the research carried out to date, there are no clear guidelines on the application, frequency, duration and periodicity of virtual reality treatments in this population, nor which are the most effective interventions for these patients. Given the importance of cancer worldwide, the high incidence it represents and the need for effective treatments that improve the quality of life of these patients, we propose a systematic review of the most updated literature in recent years to propose effective treatment alternatives that, integrated into a multidisciplinary team, help to individualize treatments to achieve the best results in cancer patients who undergo chemotherapy. Thus, the aim of this study is to find out the effectiveness of virtual reality in patients undergoing chemotherapy, to find the best application of this therapy analyzing the symptoms that could benefit from this treatment and finally, to find out patient satisfaction in relation to this treatment modality.

## 2 METHODS

### 2.1 Research strategy

To carry out this systematic review, several bibliographic searches were carried out between November 2023 and January 2024 in different databases such as Pubmed, Web of Science and PEDro. The search was conducted by combining the keywords “virtual reality,” “cancer,” “oncology,” “exercise” and “chemotherapy” (Table 1). In addition, to add more specificity to the search, the filters “last 10 years” and “randomized clinical trials” were used. Language was limited to articles published in English and Spanish. The PICO strategy was followed as follows:

- *Population*: cancer patients undergoing chemotherapy treatment.
- *Intervention*: virtual reality techniques.
- *Comparison*: conventional treatment or placebo applied in the selected clinical trials.
- *Outcomes*: effectiveness of the technique in relation to pain, anxiety and quality of life, as well as the treatment guidelines established in the prescribed treatments.

**TABLE 1:** Searches.

Database	Combination
**PUBMED**	((((virtual reality) AND (chemotherapy)) AND (treatment)) AND (oncology)) AND (cancer) ((virtual reality) AND (cancer)) AND (oncology) ((virtual reality) AND (chemotherapy)) AND (cancer) (virtual reality) AND (cancer) (virtual reality) AND (chemotherapy) (((cancer) AND (treatment)) AND (chemotherapy)) AND (exercise) (((oncology) AND (treatment)) AND (chemotherapy)) AND (exercise)
**PEDRO**	Virtual reality AND cancer
	Virtual reality AND oncology
**Web of Science**	virtual reality AND oncology AND exercise (19) all fields
	virtual reality AND cancer AND treatment AND chemotherapy (all fields)

### 2.2 Selection criteria

When selecting the studies to be included, inclusion and exclusion criteria were established. The inclusion criteria selected for this systematic review were patients with a diagnosis of cancer who were receiving chemotherapy treatment and articles and manuscripts using virtual reality techniques as treatment techniques during the chemotherapy session.

The following exclusion criteria were established: Manuscripts using virtual reality in a population other than oncology; patients without a chemotherapy prescription; associated pathologies that could interfere with the results; theoretical articles without clinical intervention; impossibility of locating the article in full text; articles in a language other than Spanish or English and other types of articles such as reviews, letters to the editor or case studies.

### 2.3 Screening, selection and extraction of data

For the selection of the sample, several search combinations with the mentioned keywords were carried out in the three selected databases. The searches can be seen in Table 1.

To obtain the final sample for this review, two researchers conducted the analysis of the manuscripts independently. Initially, prior to screening the articles, the articles that appeared duplicated were eliminated. Next, the title and abstract of each article were read according to the established inclusion criteria. With the resulting sample, both researchers analyzed the full text to select the final articles to be included as the final sample of the review. A third researcher was always available to resolve any discrepancies that arose and to reach consensus.

The extracted data were organized in a table based on author/s, year, intervention, results and conclusions to establish comparisons between them.

### 2.4 Analysis of methodological quality and risk of bias

To assess the methodological quality and risk of bias of each independently analyzed study, the PEDro scale for clinical trials was used. This scale consists of items assessing sample selection, randomization, blinding of both participants and therapists, baseline homogeneity of groups and statistical analysis. The total score is 10 points, with scores higher than 6 points being considered as high‐quality articles^28^ (Supplementary material).

## 3 RESULTS

### 3.1 Study selection

A total of 641 manuscripts were selected as potential for inclusion in the review. 65 articles were eliminated as duplicates. After screening by title and abstract, the sample consisted of 62 articles, and after independent full‐text reading by the two researchers of the resulting articles, 6 articles made up the final sample of the review.

**FIGURE 1:**
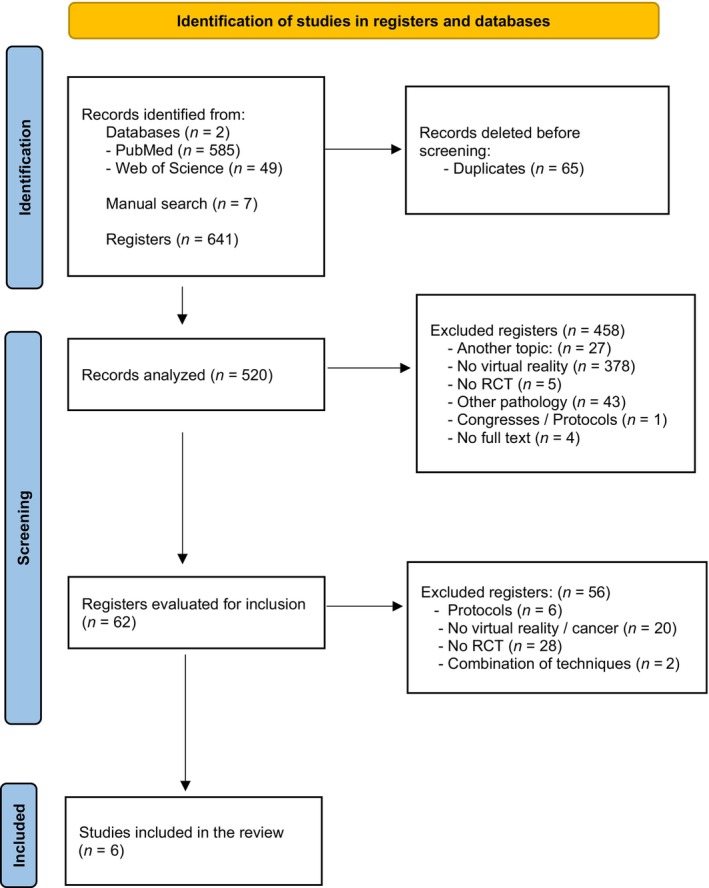
PRISMA flowchart of item selection.

Figure 1 shows the flow chart for the selection of articles.

The most relevant characteristics of each of the articles included were compiled in a table (Table 2).

**TABLE 2:** Summary of the main features of the selected articles.

Author	Sample	Variables	Procedure	Results/conclusions
Burrai et al. (2023)^29^	74 Cancer patients	Anxiety fatigue pain	Three groups: • virtual (30 min of videos with sound) • narrative medicine (30 min of writing) • general care (free activity). Three interventions administered at the beginning of treatment	Virtual reality is effective in reducing anxiety and fatigue. It did not demonstrate changes in relation to pain
Santana et al. (2023)^30^	52 women cervical cancer with radiotherapy	Anxiety	Control and experimental group: weekly chemotherapy; 5 teletherapy sessions/month and 4 insertions of brachytherapy per month. In addition, the experimental group received 12 relaxation sessions guided, 3 times/week).	Virtual reality significantly reduced anxiety in the experimental group
Reynolds et al. (2022)^31^	38 women breast cancer	Fatigue pain depression anxiety stress	“Pico Goblin Virtual Reality Headset” for home use. 2 Virtual Reality Interventions: • “Happy Place” • “Ripple” Every day, 1 week each intervention	Both interventions achieved a reduction of fatigue and improved quality of life, with duration of effects of at least 2 days
Fabi et al. (2022)^32^	44 women breast or ovarian cancer	Stress Anxiety quality of life	Experimental group: • Virtual Reality as a distraction • Control group: listening to music, watching TV, reading a newspaper, book, magazine or do nothing. Both during the first session of chemotherapy.	Anxiety was reduced in both groups being significant only in the Virtual Reality group
Wilson et al. (2021)^33^	22 men and women with cancer with chemotherapy	State of mind pain	Virtual Reality (26 scenarios natural scenarios of 2–30 min duration with music)	The reduction of pain during the chemotherapy through virtual reality was not conclusive.
Chirico et al. (2020)^34^	94 women breast cancer	Anxiety depression fatigue	• Experimental group with virtual reality (20 min) • Experimental group with music therapy (20 min of relaxing music) • Control group (free activity: reading, conversation…) During the application of chemotherapy	Virtual reality has proved to be more effective than music therapy in relieving anxiety, depression and fatigue in oncology patients

All the selected articles implemented a virtual reality program in cancer patients undergoing chemotherapy treatment. Specifically, Burrai et al.^29^ divided their sample (74 patients with a mean age of 59 years, mostly women with breast cancer in 47.2%) into three groups: one virtual reality group with Oculus Quest 2 HMD, one narrative medicine group and a third group with general care. All three interventions were performed right at the start of therapy, with the patient seated for 30 min and with a nurse available always for any needs they might have. Participants included in the first group were passive observers in several scenarios and could navigate between them by using a joystick. The scenarios were included in a youtube channel with a total of 310 videos grouped into nine categories (Africa; hills; rivers, lakes and waterfalls; islands; desert; beaches; mountains; sea and underwater environment). The sensation was not only visual but also acoustic to increase the sensations and enhance the sensory experience through headphones. On the other hand, group 2, narrative medicine, consisted of exposing, collecting and interpreting the patients' experiences of their illness with the aim of reworking their own experience by implementing reparative and beneficial mechanisms. This process could be done through writing, oral narration, poetry, drawing or even photography, being writing the one selected in this case. Finally, the third group received usual care, being free to choose the activity that the patients would do during therapy. Possible activities included talking to healthcare staff, writing, watching TV, reading, watching videos from their mobile phone or listening to music. The participants' experience was satisfactory, and they reflect that there were no possible risks during implementation. The results of the present study were not totally significant, despite not showing any change in relation to the pain presented by the patients, anxiety and fatigue decreased in the first 2 groups, being more significant with the application of virtual reality, demonstrating that this technique is an excellent distraction to reduce the stress of patients during chemotherapy sessions.

Similarly, Chirico et al.^34^ present a study with 94 women diagnosed with breast cancer with an average age around 55.5 years. In this study, they used the same 3‐group structure. In this case, in the first group, the participants used virtual reality for 5–10 min prior to the chemotherapy session to familiarize themselves with the technique and then the chemotherapy was applied. Thus, virtual reality was used for 20 min during the chemotherapy session which lasted 45–90 min. Another group had a music therapy session, in this case patients were given an mp3 player and headphones 5 min into the chemotherapy session. Again, patients listened to relaxing music prepared by an expert therapist for 20 min. Finally, the control group could choose a free activity including reading or conversation during the chemotherapy session. In this study, the occurrence of possible adverse effects such as dizziness related to the use of virtual reality was less than 20%. The results were similar for both the virtual reality group and the group receiving the music therapy intervention, but virtual reality demonstrated greater effects in relation to decreased anxiety, depression and fatigue. However, the limitations reflected by the authors such as lack of blinding and lack of randomization and the single short‐term assessment with only 2 single measurements (pre‐intervention and post‐intervention) with only one session may influence the power of the results obtained.

The research by Reynolds et al.^31^ involved 38 women with metastatic breast cancer with an average age of 52 years. Participants used the Pico Goblin VR headset together with a headset at home, instructions and questionnaires to complete. They completed a study in which virtual reality was applied in two interventions with two different modalities “Happy place” and “Ripple.” Both interventions were separated by a period of 7 days to avoid the summation of effects. The “Happy place” intervention consisted of a quiet camping scene with animation, where participants could interact with various task options, relaxation or simply listening to music. On the other hand, the intervention “Ripple” was composed of three natural environments consisting of a beach where letters can be written on the beach or in the sky, a waterfall where the participant can stack stones or a mountain range where the participant can jump between peaks and lakes. Participants were asked to use the present technology every day for at least 10 min and to fill in a follow‐up sheet to assess adherence to the treatment. The assessment questionnaires were completed online and at six different points (pre‐intervention, post‐intervention of each of the interventions and a follow‐up after each intervention). The results presented by these authors were a high acceptance by the participants, although some reported adverse effects such as feeling claustrophobia, a little dizziness or even nausea, achieving with both interventions a reduction in fatigue, pain, depression, anxiety and stress, which, although not sufficient to demonstrate clinical changes, have shown beneficial effects improving the quality of life. The limitations of the present study were again the lack of randomization, as the participants were divided into two groups under the premise of being over or under 50 years of age to establish the order of starting with each modality, although both groups carried out the two interventions, and the lack of a control group. The short use of this technique (only 1 week with each intervention) should also be considered, although it is longer than other studies in which only a single session is applied.

Along these lines, Wilson et al.^33^ also applied the virtual reality technique in a convenience sample of 22 subjects, men (41%) and women (59%) receiving chemotherapy with a mean age of 61 years. Prior to the start of the intervention, participants completed questionnaires regarding their pain and mood using the STAI questionnaire. Subsequently, the virtual reality intervention consisted of a variety of 26 natural scenarios with relaxing music that could be used for 2–30 min while the patient was seated receiving their chemotherapy session. Participants could stop the intervention at any time. After completion, participants were reassessed. Limitations presented by the researchers reflect the difficulty in recruiting the sample due to the pandemic period, as well as the lack of a control group. The results obtained show a reduction in pain during the chemotherapy session and despite inconclusive results, participants reported a decrease in tension, an increase in feelings of calm, relaxation and well‐being. In addition, none of the participants experienced cybernetics‐related adverse effects such as nausea or vomiting, dizziness, headache or even visual problems or disturbances.

The other authors also applied virtual reality to one of the groups; in this case, Fabi et al.^32^ carried out a study with 44 women diagnosed with breast or ovarian cancer with an average age of around 51 years and who were going to receive the first cycle of chemotherapy treatment. In this study, all patients received training to familiarize them with the virtual reality technique and how to select their preferred content. The content was selected by the researchers based on comfort during viewing to avoid sudden movements that could cause adverse effects such as nausea or vomiting; that the content was attractive and relaxing for the users (walks through the capitals of European cities, concerts, natural mountain environments, isolated places, beaches or even yoga sessions) and finally, that the duration and time that the patients would be viewing the content was comfortable, never exceeding 10 min and being able to choose between 3 and 4 contents for each patient. The application of virtual reality started when the chemotherapy therapy began and lasted for a maximum of 60–90 min. While the experimental group received virtual reality therapy, the control group received distractions such as reading, listening to music or talking, both during the chemotherapy session. The results showed that anxiety was only significantly reduced in the experimental group with virtual reality. Again, the limitations agreed with the other authors, the small sample size and the short‐term analysis are shown to be the main ones. Perhaps the fact that it was used prior to the first cycle of chemotherapy could influence the results obtained, being more effective in the use of virtual reality during clinical practice.

Finally, Santana et al.^30^ presented a study with 52 women diagnosed with cervical cancer with a mean age of 43.2 years, who were to receive chemotherapy and radiation treatment. Both groups received a treatment duration of 28 days; the conventional treatment consisted of weekly chemotherapy sessions and 4 brachytherapy sessions per month. The experimental group underwent 12 virtual reality sessions in chemotherapy and brachytherapy addiction, three times a week. Participants sat in a quiet room with a comfortable seat and could choose from a variety of videos (sunsets, floating on water, sitting on the beach or floating in space). All these videos were 9 min and 52 s long. The videos contained an introduction of 5 min and 25 s with a black screen to induce a state of relaxation along with an audio that guided breathing; then for 4 min and 14 s, the images continued with immersion in nature, interacting with smells, sounds, textures and temperatures; sensations that could be alternated with slow and deep breathing. In the last 13 s of the video, the participant was invited to focus on the sensations, the rhythm of her breathing, to calmly return to reality. Limitations noted by the authors were the absence of blindness; however, a strength of this study was that all interventions were conducted by the same researcher to ensure the same protocol for all subjects. The results showed a significant reduction in anxiety in the experimental group, although there was no assessment of the safety of the intervention, the absence of dropouts and adverse effects, postulate this technique as a feasible and easy to apply technique.

## 4 DISCUSSION

Virtual reality has proven to be an effective technique in reducing the anxiety, pain, asthenia and stress suffered by patients diagnosed with cancer and chemotherapy treatment. The distraction that this therapeutic modality generates helps to reduce the perception of pain and worry that these procedures generate. However, there are no standard application guidelines or application protocols that demonstrate the superiority of one technique over another.

The main complication in establishing these guidelines lies in sample selection; most authors present a convenience sample,^33^ they did not randomize the sample.^31^, ^34^ In addition, some authors had their intervention suspended due to the Covid‐19 pandemic.^30^

Another aspect shared by several authors is the great variety of scenarios they offer, thus being able to individualize treatments and guaranteeing care, since it is the patient himself who decides the scenario he wishes to visualize^29^; however, this may entail a limitation in relation to the external validity of the results since it is not possible to establish comparisons if different visualizations have been carried out or with different durations. Thus, Santana et al.^30^ protocolized the intervention for all patients who participated in the study, with the same therapist guiding and orienting the sessions to ensure that all participants had the same intervention and the same duration. Most of the scenarios chosen by the authors^29^, ^31^ are natural spaces such as rivers, lakes, sea, mountains or beaches, among others. It seems that previous studies on this aspect have corroborated that these natural environments have been shown to generate a sense of peace and relaxation.^35^ In addition, virtual reality in natural environments has also been shown to have an analgesic effect. This is due to the shift of attention from the painful stimulus, in this case chemotherapy, to more pleasant sensations, which reduces the perception of pain at the conscious level.^36^

The duration of the interventions is another controversial aspect on which there is no agreement, as they can last from 2^33^ to 90 min,^32^ which makes it difficult to establish a protocol or standardization of the procedures. Burrai et al.^29^ established a virtual reality intervention with a duration of 30 min while other authors such as Chirico et al.^34^ proposed a slightly shorter duration with 20 min of application and Reynolds et al.^31^ further decreased this guideline by establishing a minimum of at least 10 min. However, other authors could not establish a specific duration as patients were free to interrupt the intervention, change the video or keep it, providing flexibility and individualization to the treatment.^33^

There are also a variety of virtual reality modalities. In the case of the study by Burrai et al.,^29^ patients did not perform any intervention in the scenarios they visualized, the intervention consisted of a contemplative immersion modality with participants being mere passive spectators of the landscape or natural environment they had selected. This may be less effective than interventions that do involve active participation by the patient in the therapy.^29^ The opposite case was implemented by Santana et al.^30^ who proposed an intervention where participants could interact with smells, textures, temperature and even guide breathing; similarly Reynolds et al.^31^ proposed two virtual reality modalities, one of them being “Happy place” where three different scenarios were presented where participants could write letters on the beach or in the sky, a waterfall where the participant could stack stones or a mountain range where the participant can jump between peaks and lakes.

Regarding frequency, most studies only present a single session of virtual reality or other interventions in a single chemotherapy session, without evaluating the summative effect that this therapy could have when applied periodically or even at home to enhance its effect, which may be a limitation in the use of this virtual reality technique^29^; this aspect has been reflected as a limitation, exposing as a future line of intervention the need for more virtual reality sessions; however, they also expose the possibility of loss of effectiveness if there is a habituation to this treatment in the face of medical procedures.^34^ This limitation was covered by Reynolds et al.^31^ who implemented a home treatment for 7 days (at least 10 min per day) and Santana et al.^30^ who scheduled an intervention of three sessions per week for 28 days. Despite these results, the need for more durable treatments with long‐term follow‐up is necessary to know in a more objective way the extent of this therapeutic modality in cancer patients receiving chemotherapy treatment.

In summary, the heterogeneity of the results found could be due to the differences in samples and the lack of consensus regarding the number of sessions, frequency, periodicity, type of intervention/virtual reality modality or duration of follow‐up to obtain statistically significant results to support a standard guideline for this therapy.

The studies selected in this review have carried out virtual reality interventions during chemotherapy sessions, either during the whole chemotherapy session or during part of it, except for the study by Reynolds et al.,^31^ who emphasize the importance of homework, with the patient being able to choose the time when he or she wishes or feels better to carry out the treatment intervention. On the other hand, it again limits standardization, as follow‐up or adherence may be compromised as the exact treatment indication to be performed is not prescribed. This has already been postulated by Espinoza et al.,^27^ who highlighted virtual reality as a positive therapy that can be used to promote well‐being even in hospitalized patients, and therefore propose the need to carry out these interventions in patients who are hospitalized to generate distracting situations from the medical procedures they have been prescribed or from the painful processes of the treatments they are receiving in order to improve the symptomatology they are suffering from. They carried out a study with 33 hospitalized patients to measure depression and anxiety in four 30‐min sessions over the course of a week. During the interventions they used two environments to generate joy and relaxation, achieving a significant decrease in stress levels by increasing positive emotions, which demonstrates the effectiveness of this technique in this type of population. Reviewing the literature, there are several publications that have presented promising preliminary results during the implementation of a clinical trial, with virtual reality being feasible with short sessions in the patient's own room, which can be adapted to the patients' presenting routines without interfering with their consultations and/or treatments^27^; unfortunately, no definitive results have yet been presented to support or refute these interventions. Similarly, several protocols have been found that have subsequently failed to fully finalize the interventions.

Most authors set up several treatment groups to test the superiority of virtual reality over other techniques such as narrative medicine,^29^ music therapy,^34^ reading, listening to music or talking to healthcare staff.^32^ Other authors complemented conventional treatment (chemotherapy and brachytherapy) with virtual reality.^30^ Although no statistically significant results have been obtained to support the choice of virtual reality over other treatment techniques, the results are positive and promising, and it has been postulated as a complement to conventional therapies.

In relation to the symptoms experienced by patients, there is controversy. On the one hand, cancer patients may experience a wide variety of symptoms that affect and interfere with the quality of life; however, the timing of treatment may influence the perception of these symptoms, in which case there is a difference between interventions during the chemotherapy session,^29^ before starting the first cycle^32^ or from home, with the intervention being carried out at the time of their choice.^31^

Besides, virtual reality has been used in populations other than adult cancer patients. Specifically, this technique has also been used in other populations such as children and adolescents with cancer and in other procedures such as injections, cures, burns or even physiotherapy treatment, reducing the perception of fear and obtaining a positive sensation that facilitates the performance of these interventions.^37^, ^38^, ^39^

Similarly, virtual reality has been used in other non‐cancer pathologies, for example, in an article published by Lewandowski et al.^40^ in which virtual reality therapy was used to reduce stress and anxiety in patients with inflammatory bowel disease. In this article, the intervention was performed on 90 patients divided into an experimental group with immersive virtual reality for 15 min in the hospital itself and a control group with treatment as usual. This therapy has even been used to improve patient education about prescribed procedures or techniques. Marqués et al.^41^ demonstrated this in their study where they managed to reduce anxiety and improve understanding of their treatment by using an educational tool in a virtual environment like a flight simulator with 32 prostate cancer patients. These techniques not only improve patient satisfaction with their treatment but also improve the quality of healthcare systems.

This review has several limitations, including strict inclusion and exclusion criteria that may have led to some bias in the selection of articles and the publication of these results. The limited sample size of the selected studies, as well as the lack of randomization in some of them, the diversity of chemotherapy treatments and tumors treated, prevent extrapolation of the results. Furthermore, there is a clear predominance of women in the studies analyzed, which it should be considered because of the possible differences between sexes in relation to the symptoms analyzed as several authors have been stated in relation of anxiety^42^ or stress.^43^ Finally, the difference in the type of tumor or even the differences in the medication taken by the participants could have influenced the results obtained.

## 5 CONCLUSION

Virtual reality could be a valid complementary tool in the treatment of patients undergoing chemotherapy. The studies analyzed show that virtual reality shows positive results in pain reduction, anxiety, stress and asthenia. However, more studies are needed, with larger sample sizes and long‐term follow‐ups to establish treatment protocols in relation to the frequency, intensity, duration and periodicity and modality of interventions with virtual reality.

## AUTHOR CONTRIBUTIONS

**Jorge Juan Alvarado‐Omenat:** Conceptualization; investigation; methodology; writing – review and editing; writing – original draft. **Rocío Llamas‐Ramos:** Investigation; conceptualization; writing – original draft; writing – review and editing; methodology. **Daniel García‐García:** Investigation; writing – review and editing. **Marta Correyero‐León:** Investigation; writing – review and editing. **Emilio Fonseca‐Sánchez:** Conceptualization; supervision; methodology; investigation; writing – review and editing. **Inés Llamas‐Ramos:** Conceptualization; investigation; writing – original draft; writing – review and editing; methodology; project administration; supervision.

## CONFLICT OF INTEREST STATEMENT

No financial or non‐financial benefits have been received or will be received from any party related directly or indirectly to the subject of this article.

## Supporting information


Data S1.

## Data Availability

The datasets generated and/or analyzed during the current study are available from the corresponding author on reasonable request.
