# Supplementary material for: Effectiveness of virtual reality in cancer patients undergoing chemotherapy. Systematic review
Source: Int J Cancer. 2024 Nov 16;156(7):1419–28. doi: 10.1002/ijc.35258 (PMC11789450; doi:10.1002/ijc.35258)
Supplement: Supplementary file 1 — Data S1. [file IJC-156-1419-s001.pdf]

Supplementary materials & methods

EFFECTIVENESS OF VIRTUAL REALITY IN CANCER PATIENTS UNDERGOING CHEMOTHERAPY. SYSTEMATIC REVIEW

Authors: Jorge Juan Alvarado-Omenat, Rocío Llamas-Ramos, Daniel García-García, Marta Correyero-León, Emilio Fonseca-Sánchez, Inés Llamas-Ramos

TABLE OF CONTENT

- PEDro scale

Escala PEDro-Español

1.	Los criterios de elección fueron especificados	no ❑ si ❑	donde:
2.	Los sujetos fueron asignados al azar a los grupos (en un estudio cruzado, los sujetos fueron distribuidos aleatoriamente a medida que recibían los tratamientos)	no ❑ si ❑	donde:
3.	La asignación fue oculta	no ❑ si ❑	donde:
4.	Los grupos fueron similares al inicio en relación a los indicadores de pronostico más importantes	no ❑ si ❑	donde:
5.	Todos los sujetos fueron cegados	no ❑ si ❑	donde:
6.	Todos los terapeutas que administraron la terapia fueron cegados	no ❑ si ❑	donde:
7.	Todos los evaluadores que midieron al menos un resultado clave fueron cegados	no ❑ si ❑	donde:
8.	Las medidas de al menos uno de los resultados clave fueron obtenidas de más del 85% de los sujetos inicialmente asignados a los grupos	no ❑ si ❑	donde:
9.	Se presentaron resultados de todos los sujetos que recibieron tratamiento o fueron asignados al grupo control, o cuando esto no pudo ser, los datos para al menos un resultado clave fueron analizados por "intención de tratar"	no ❑ si ❑	donde:
10.	Los resultados de comparaciones estadísticas entre grupos fueron informados para al menos un resultado clave	no ❑ si ❑	donde:
11.	El estudio proporciona medidas puntuales y de variabilidad para al menos un resultado clave	no ❑ si ❑	donde:

La escala PEDro está basada en la lista Delphi desarrollada por Verhagen y colaboradores en el Departamento de Epidemiología, Universidad de Maastricht (*Verhagen AP et al (1998). The Delphi list: a criteria list for quality assessment of randomised clinical trials for conducting systematic reviews developed by Delphi consensus. Journal of Clinical Epidemiology, 51(12):1235-41*). En su mayor parte, la lista está basada en el consenso de expertos y no en datos empíricos. Dos ítems que no formaban parte de la lista Delphi han sido incluidos en la escala PEDro (ítems 8 y 10). Conforme se obtengan más datos empíricos, será posible "ponderar" los ítems de la escala, de modo que la puntuación en la escala PEDro refleje la importancia de cada ítem individual en la escala.

El propósito de la escala PEDro es ayudar a los usuarios de la bases de datos PEDro a identificar con rapidez cuales de los ensayos clínicos aleatorios (ej. RCTs o CCTs) pueden tener suficiente validez interna (criterios 2-9) y suficiente información estadística para hacer que sus resultados sean interpretables (criterios 10-11). Un criterio adicional (criterio 1) que se relaciona con la validez externa ("generalizabilidad" o "aplicabilidad" del ensayo) ha sido retenido de forma que la lista Delphi esté completa, pero este criterio no se utilizará para el cálculo de la puntuación de la escala PEDro reportada en el sitio web de PEDro.

La escala PEDro no debería utilizarse como una medida de la "validez" de las conclusiones de un estudio. En especial, avisamos a los usuarios de la escala PEDro que los estudios que muestran efectos de tratamiento significativos y que puntúen alto en la escala PEDro, no necesariamente proporcionan evidencia de que el tratamiento es clínicamente útil. Otras consideraciones adicionales deben hacerse para decidir si el efecto del tratamiento fue lo suficientemente elevado como para ser considerado clínicamente relevante, si sus efectos positivos superan a los negativos y si el tratamiento es costo-efectivo. La escala no debería utilizarse para comparar la "calidad" de ensayos realizados en las diferentes áreas de la terapia, básicamente porque no es posible cumplir con todos los ítems de la escala en algunas áreas de la práctica de la fisioterapia.

Notas sobre la administración de la escala PEDro:

Todos los criterios	**<u>Los puntos solo se otorgan cuando el criterio se cumple claramente</u>**. Si después de una lectura exhaustiva del estudio no se cumple algún criterio, no se debería otorgar la puntuación para ese criterio.
Criterio 1	Este criterio se cumple si el artículo describe la fuente de obtención de los sujetos y un listado de los criterios que tienen que cumplir para que puedan ser incluidos en el estudio.
Criterio 2	Se considera que un estudio ha usado una designación al azar si el artículo aporta que la asignación fue aleatoria. El método preciso de aleatorizacion no precisa ser especificado. Procedimientos tales como lanzar monedas y tirar los dados deberían ser considerados aleatorios. Procedimientos de asignación cuasi-aleatorios, tales como la asignación por el número de registro del hospital o la fecha de nacimiento, o la alternancia, no cumplen este criterio.
Criterio 3	*La asignación oculta* (enmascaramiento) significa que la persona que determina si un sujeto es susceptible de ser incluido en un estudio, desconocía a que grupo iba a ser asignado cuando se tomó esta decisión. Se puntúa este criterio incluso si no se aporta que la asignación fue oculta, cuando el artículo aporta que la asignación fue por sobres opacos sellados o que la distribución fue realizada por el encargado de organizar la distribución, quien estaba fuera o aislado del resto del equipo de investigadores.
Criterio 4	Como mínimo, en estudios de intervenciones terapéuticas, el artículo debe describir al menos una medida de la severidad de la condición tratada y al menos una medida (diferente) del resultado clave al inicio. El evaluador debe asegurarse de que los resultados de los grupos no difieran en la línea base, en una cantidad clínicamente significativa. El criterio se cumple incluso si solo se presentan los datos iniciales de los sujetos que finalizaron el estudio.
Criterio 4, 7-11	*Los Resultados clave* son aquellos que proporcionan la medida primaria de la eficacia (o ausencia de eficacia) de la terapia. En la mayoría de los estudios, se usa más de una variable como una medida de resultado.
Criterio 5-7	*Cegado* significa que la persona en cuestión (sujeto, terapeuta o evaluador) no conocía a que grupo había sido asignado el sujeto. Además, los sujetos o terapeutas solo se consideran "cegados" si se puede considerar que no han distinguido entre los tratamientos aplicados a diferentes grupos. En los estudios en los que los resultados clave sean auto administrados (ej. escala visual analógica, diario del dolor), el evaluador es considerado cegado si el sujeto fue cegado.
Criterio 8	Este criterio solo se cumple si el artículo aporta explícitamente *tanto* el número de sujetos inicialmente asignados a los grupos *como* el número de sujetos de los que se obtuvieron las medidas de resultado clave. En los estudios en los que los resultados se han medido en diferentes momentos en el tiempo, un resultado clave debe haber sido medido en más del 85% de los sujetos en alguno de estos momentos.
Criterio 9	El análisis por *intención de tratar* significa que, donde los sujetos no recibieron tratamiento (o la condición de control) según fueron asignados, y donde las medidas de los resultados estuvieron disponibles, el análisis se realizó como si los sujetos recibieran el tratamiento (o la condición de control) al que fueron asignados. Este criterio se cumple, incluso si no hay mención de análisis por intención de tratar, si el informe establece explícitamente que todos los sujetos recibieron el tratamiento o la condición de control según fueron asignados.
Criterio 10	Una comparación estadística *entre grupos* implica la comparación estadística de un grupo con otro. Dependiendo del diseño del estudio, puede implicar la comparación de dos o más tratamientos, o la comparación de un tratamiento con una condición de control. El análisis puede ser una comparación simple de los resultados medidos después del tratamiento administrado, o una comparación del cambio experimentado por un grupo con el cambio del otro grupo (cuando se ha utilizado un análisis factorial de la varianza para analizar los datos, estos últimos son a menudo aportados como una interacción grupo x tiempo). La comparación puede realizarse mediante un contraste de hipótesis (que proporciona un valor "p", que describe la probabilidad con la que los grupos difieran sólo por el azar) o como una estimación de un tamaño del efecto (por ejemplo, la diferencia en la media o mediana, o una diferencia en las proporciones, o en el número necesario para tratar, o un riesgo relativo o hazard ratio) y su intervalo de confianza.
Criterio 11	Una *estimación puntual* es una medida del tamaño del efecto del tratamiento. El efecto del tratamiento debe ser descrito como la diferencia en los resultados de los grupos, o como el resultado en (cada uno) de todos los grupos. Las *medidas de la variabilidad* incluyen desviaciones estándar, errores estándar, intervalos de confianza, rango intercuartílicos (u otros rangos de cuantiles), y rangos. Las estimaciones puntuales y/o las medidas de variabilidad deben ser proporcionadas gráficamente (por ejemplo, se pueden presentar desviaciones estándar como barras de error en una figura) siempre que sea necesario para aclarar lo que se está mostrando (por ejemplo, mientras quede claro si las barras de error representan las desviaciones estándar o el error estándar). Cuando los resultados son categóricos, este criterio se cumple si se presenta el número de sujetos en cada categoría para cada grupo.
